# Severe acute myositis and myocarditis on initiation of 6-weekly pembrolizumab post-COVID-19 mRNA vaccination

**DOI:** 10.1136/jitc-2023-008151

**Published:** 2024-04-24

**Authors:** Robert A Watson, Weiyu Ye, Chelsea A Taylor, Elsita Jungkurth, Rosalin Cooper, Orion Tong, Tim James, Brian Shine, Monika Hofer, Damian Jenkins, Robert Pell, Eleni Ieremia, Stephanie Jones, David Maldonado-Perez, Ian S D Roberts, Nicholas Coupe, Mark R Middleton, Miranda J Payne, Benjamin P Fairfax

**Affiliations:** 1 MRC Weatherall Institute of Molecular Medicine, University of Oxford, Oxford, UK; 2 Department of Oncology, University of Oxford, Oxford, UK; 3 Cancer and Haematology Centre, Oxford University Hospitals NHS Foundation Trust, Oxford, UK; 4 Department of Clinical Biochemistry, Oxford University Hospitals NHS Foundation Trust, Oxford, UK; 5 Department of Neuro Pathology, Oxford University Hospitals NHS Foundation Trust, Oxford, UK; 6 Department of Clinical Neurology, Oxford University Hospitals NHS Foundation Trust, Oxford, UK; 7 Department of Cellular Pathology, Oxford University Hospitals NHS Foundation Trust, Oxford, UK; 8 Oxford Centre for Histopathological Research, Oxford University Hospitals NHS Trust, Oxford, UK

**Keywords:** Case Reports, Immune Checkpoint Inhibitors, Melanoma, Receptors, Antigen, T-Lymphocytes

## Abstract

We describe three cases of critical acute myositis with myocarditis occurring within 22 days of each other at a single institution, all within 1 month of receiving the initial cycle of the anti-PD-1 drug pembrolizumab. Analysis of T cell receptor repertoires from peripheral blood and tissues revealed a high degree of clonal expansion and public clones between cases, with several T cell clones expanded within the skeletal muscle putatively recognizing viral epitopes. All patients had recently received a COVID-19 mRNA booster vaccine prior to treatment and were positive for SARS-CoV2 Spike antibody. In conclusion, we report a series of unusually severe myositis and myocarditis following PD-1 blockade and the COVID-19 mRNA vaccination.

## Insights

We report a cluster of three cases, within a 3-week window, of severe myocarditis with myositis occurring post cycle 1 of PD-1 blockade after COVID-19 booster vaccination—a high degree of T cell repertoire overlap between cases was observed, suggestive of sharing of autoantigen reactivity.

## Introduction

Immune checkpoint blockade (ICB) with anti-PD-1 monoclonal antibodies is approved for treatment of melanoma in adjuvant and palliative settings.^1 2^ These treatments can elicit immune-related adverse events (irAEs)^3 4^ including myositis and myocarditis—although the occurrence of these particular toxicities is very rare, being observed in <1% of cases with fatalities reported in <0.01% of recipients of anti-PD-1 alone.^5 6^

Infection with SARS-CoV2 can lead to both myositis and myocarditis.^7^ While the underlying mechanisms remain undetermined, indirect virally triggered autoimmune reaction or direct epitope cross-reactivity are posited.^8^ There is also an association between SARS-CoV2 vaccination with mRNA vaccines and myocarditis. This is rare, appears to be driven by a younger patient population and the underlying mechanisms are currently unclear.^9–12^

## Case reports

### Patient 1

**Figure 1:**
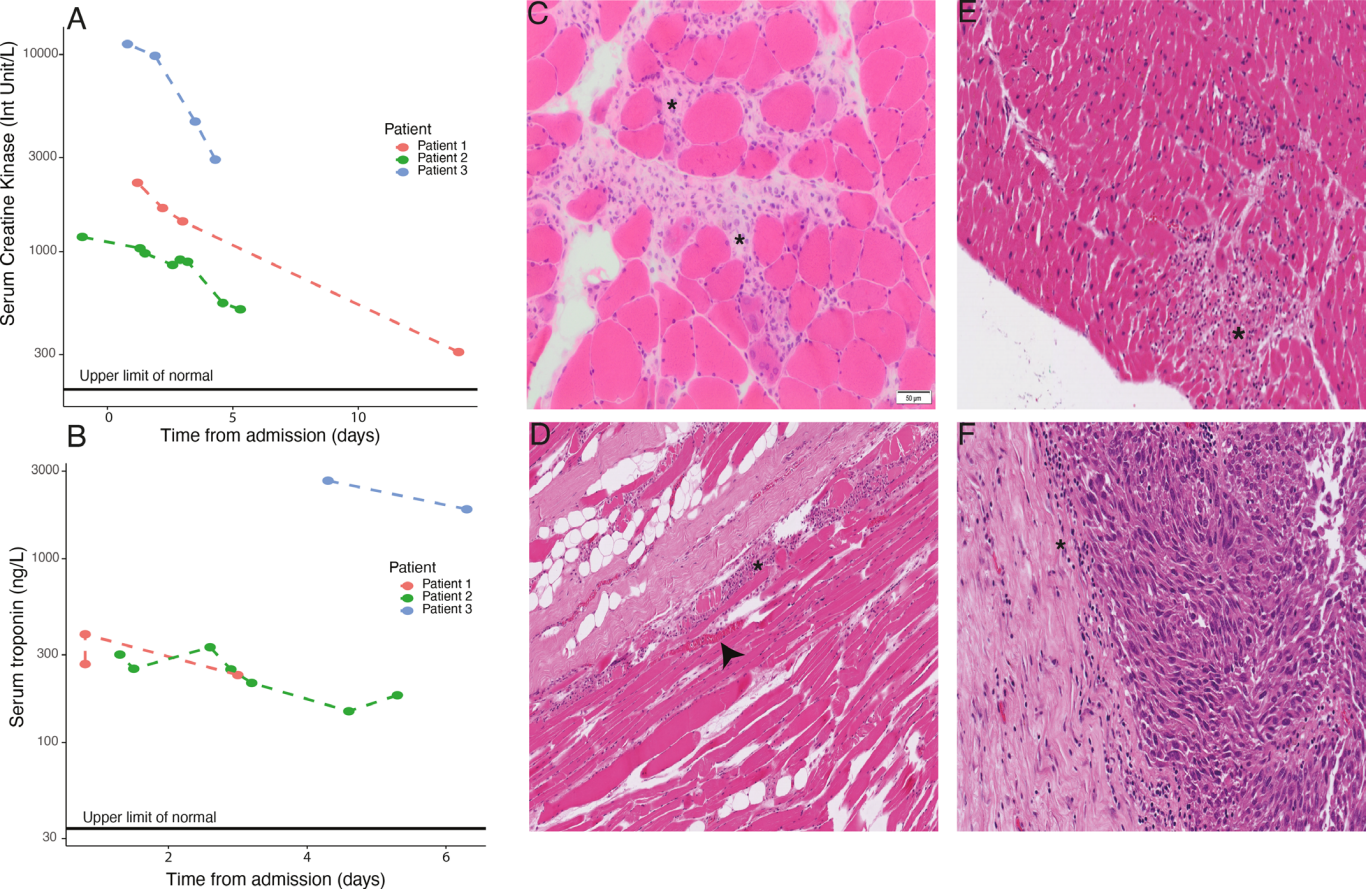
(A) Serum creatine kinase (CK) by day postadmission for each of the three patients. (B) as per (A) but for troponin. (C) H&E slide of fresh skeletal muscle biopsy taken from patient 1. Widespread leucocyte infiltration can be seen (eg, at asterixes). (D) H&E-stained slide of postmortem (PM) skeletal muscle sample taken from patient 3. The asterix indicates infiltrating lymphocytes with the arrowhead denoting myocyte necrosis. (E) H&E slide of PM cardiac muscle from patient 3, demonstrating widespread leucocyte infiltration. (F) As per (E) but for tumor deposit taken from the small bowel serosa.

A patient in their early 70s with pretreatment Eastern Cooperative Oncology Group (ECOG) performance status (PS) 0 presented with dizziness and dyspnea 28 days postinitiation of pembrolizumab (6-week infusion, 400 mg) for adjuvant treatment of resected stage IIIC melanoma. Medical history consisted of atrial fibrillation and type 2 diabetes. They attended a nearby hospital 5 days prior for non-specific chest pain, treated with analgesia. On arrival, they reported lethargy, weakness and inability to support their head, with associated bulbar symptoms. They had no chest pain or ischemic ECG changes, but serum creatine kinase (CK) and troponin were markedly elevated, (CK: 2236 U/L, range 30–200 U/L; troponin: 267 ng/L, range 0–34 ng/L; figure 1A,B and table 1) as were liver enzymes (alanine aminotransferase (ALT), 394 U/L, range 10–45 U/L; alkaline phosphatase (ALP), 1044 U/L, range 30–130 U/L) and bilirubin (68 umol/L, range 0–21 umol/L). Their condition acutely worsened, developing type II respiratory failure with reduced consciousness, precipitating transfer to critical care with vasopressor support and ventilation. Initial management was with intravenous methylprednisolone (2 mg/kg) for 5 days but on further deterioration, intravenous immunoglobulin was given for a further 5 days. An electromyogram demonstrated a necrotic myopathic process involving proximal limbs with background neuropathy. Autoantibody screening revealed positivity for anti-acetylcholine receptor antibodies (AChR). A muscle biopsy showed multifocal clusters of necrotic fibers, consistent with an ICB-associated myositis^13^ (figure 1C, online supplemental report 1). The final diagnosis was pembrolizumab-associated myositis, myocarditis and myasthaenia gravis with hepatitis. Pyridostigmine was commenced, with little initial benefit, followed by plasma exchange (commenced 3 weeks following intravenous immunoglobulin). A slow but sustained clinical improvement ensued and they were discharged to a rehabilitation unit. They received a booster vaccination (BNT162b2) 28 days prior to pembrolizumab.

10.1136/jitc-2023-008151.supp1 Supplementary data

**Table 1:** Summary of cases of myositis or myocarditis, comparing index cases (top section) to those found within a cohort of 237 patients from end 2015 to end 2021 (‘prior to index cases’, bottom section) and a further case identified subsequent to the index cases in early 2022

	Patient	Type	Time period	Treatment	Day post- treatment	Number of cycles received	Peak CK	Peak troponin	Received steroids?	Admitted?	Overlap
Index cases	Patient 1	Myositis with myocarditis	Late 2021	PD-1 blockade alone	28	1	2236	388	Yes	Yes	Hepatitis, myaesthenia gravis
	Patient 2	Myositis with myocarditis	Early 2022	PD-1 blockade alone	39	1	1186	2101	Yes	Yes	Colitis, Hepatitis, myaesthenia gravis (clinical)
	Patient 3	Myositis with myocarditis	Late 2021	PD-1 blockade alone	23	1	11 301	2664	Yes	Yes	Hepatitis
Prior to index cases		Myositis alone	Early 2018	PD-1 + CTLA-4 blockade	49	2	8938	N/A	Yes	Yes	Hepatitis
		Myositis alone	Late 2018	PD-1 + CTLA-4 blockade	28	2	5733	N/A	Yes	No	No
After index cases		Myositis with myocarditis	Early 2022	PD-1 + CTLA-4 blockade	43	2	1229	90	Yes	Yes	Hepatitis and nephritis

CK, creatine kinase; N/A, not available.

### Patient 2

A patient in their 80s with pretreatment PS 0 and no relevant medical history was admitted 39 days postinitiation of pembrolizumab (400 mg) for stage IV melanoma. They developed diarrhea and severe myalgia 11 days after treatment, attending a local hospital where oral prednisolone (60 mg) was commenced. Subsequently, the diarrhea resolved whereas the myalgia worsened and on day 23 they were readmitted having collapsed with loss of consciousness. They were in complete heart block but without ECG features of acute ischemia, however, serum troponin was elevated (1124 ng/L). Coronary angiography demonstrated a possible ruptured left anterior descending artery plaque and they were managed with percutaneous stent placement and a permanent pacemaker. Despite this, blood tests 6 days postdischarge showed further elevation of serum troponin (2101 ng/L) and elevated CK (1186 U/L) (figure 1A,B and table 1) which precipitated admission to our center (day 39 post-treatment). ALT was also mildly elevated (216 U/L), ALP was normal. A clinical diagnosis of immunotherapy-associated myositis and myocarditis was made and intravenous methylprednisolone was given for 3 days, followed by oral prednisolone with slow clinical and biochemical improvement. However, 10 weeks postdischarge they were readmitted with increasing fatigue, weakness, dysarthria, diplopia and ptosis. AChR antibodies were negative. A clinical diagnosis of pembrolizumab- associated ocular myopathy and myositis was made, along with possible myasthenia; management was with oral corticosteroids, a course of intravenous immunoglobulin and physiotherapy rehabilitation. They received a booster vaccination (BNT162b2) 74 days prior to pembrolizumab.

### Patient 3

A patient in their early 80s with pretreatment PS 0 was admitted with 5 days of reduced mobility, fatigue and myalgia (on movement but not palpation) 23 days postinitiation of pembrolizumab (400 mg) for stage IV melanoma. CK was markedly elevated (11 301 U/L) (figure 1A, table 1), as were liver enzymes (ALT 705 U/L, ALP 155 U/L). Troponin, measured on day 4 postadmission was 2931 ng/L (figure 1B, table 1). Management was with intravenous methylprednisolone, switched to oral prednisolone after 4 days. Supplemental oxygen was started but weaned due to symptomatic improvement and decline in CK (figure 1A) over 4 days, although bulbar symptoms and dysphagia then developed. An echocardiogram performed 5 days postadmission demonstrated normal biventricular systolic function. Six days postadmission, they acutely deteriorated with type II respiratory failure and subsequent cardiac arrest. A postmortem (PM) examination demonstrated multiple foci of inflammation and myocyte necrosis throughout the myocardium. Replacement fibrosis was absent, indicating a 2-week time frame, and there was minimal atheroma and no myocardial infarction. Skeletal muscle examination demonstrated multiple foci of inflammation and necrosis, similar to the sampled myocardium. Of note, a metastatic tumor deposit from the serosal surface of the small bowel displayed brisk lymphocyte infiltration (figure 1D,E and F, online supplemental report 2). They had received a booster vaccination (BNT162b2) 74 days prior to pembrolizumab.

All three patients were negative for SARS-CoV2 antinucleocapsid IgG and positive for antispike IgG. No patients received any subsequent ICB.

## Methods

We performed an analysis of irAEs occurring within a cohort of patients receiving ICB (both anti-PD-1 and anti-PD-1/CTLA-4 combined at varying doses) for melanoma and renal cell carcinoma since 2015.^3 14–16^

We analyzed T cell receptor (TCR) repertoires from peripheral blood and tissue taken from the three patients reported here. PM tissue was obtained with informed consent from relatives and under institutional ethical approval (CUREC-1, R80630/RE001).

Blood collected into EDTA-coated tubes was separated using density centrifugation (Ficoll Paque) with plasma removed and ultracentrifuged. Routine biochemistry tests were undertaken on thawed plasma samples using the Abbott Architect c16000; hs-troponin and COVID antibodies using an Abbott Architect i2000. Whole PBMCs and magnetically sorted CD8+T cells (MACS system, Miltenyi Biotec) were lysed in RLT Plus buffer supplemented with 40 mM DTT, homogenized using QIAshredder columns prior to RNA and gDNA extraction using AllPrep DNA/RNA/miRNA Universal extraction kits (Qiagen). Fresh skeletal muscle was flash-frozen at −80°C before RNA was extracted using the RNeasy Plus Universal Mini Kit (Qiagen). RNA was extracted from paraffin-embedded PM tissue using the AllPrep FFPE kit (Qiagen). TCR repertoire libraries were constructed using the QIAseq Immune Repertoire library kit (Qiagen). Sequencing was performed on a MiSeq (Illumina) with preprocessing and alignment using the CLC genomics workbench (Qiagen). CDR3B chains were matched to epitopes using The Immune Epitope Database TCRMatch tool (http://tools.iedb.org/tcrmatch/) with the highest scoring epitope match being assigned to each CDR3B chain. All downstream bioinformatic and statistical analyses were performed in R (V.4.0.5).

## Results

### Comparison with cohort from this center

We examined the incidence of myositis and myocarditis across a cohort of patients receiving ICB for melanoma and renal cell carcinoma from end 2015 to end 2021,^3 14–16^ (n=135 who received combination programmed cell death protein 1 (PD-1) and cytotoxic T-lymphocyte-associated antigen 4 (CTLA-4) blockade, n=102 who received single-agent anti-PD-1). In line with published incidence,^6^ only 2 of the 237 patients suffered biochemically proven myositis and no myocarditis cases were recorded. Both patients had received two doses of combined PD-1/CTLA-4 blockade (cICB) (table 1). After the occurrence of the three index cases which form this series, we identified a further individual in our cohort who developed clinical myositis with an asymptomatic troponin rise after their second cycle of cICB for metastatic renal cell cancer, 66 days postbooster (BNT162b2). This culminates in four cases of myositis with likely myocarditis within 28 days over the winter of 2021–2022, on a background of only two cases of myositis without myocarditis occurring within the same patient cohort over the preceding 6 years. In the three index cases, we screened serum for the development of muscle specific immunoreactivity with standard-of-care immuno-blots against cN-1A, MDA-5, Tif1-gamma, NXP-2, SAE-1, Mi-2a, Mi-2b, Ku, PM-Scl 100, PM-Scl-75, Jo-1, SRP, PL-7, PL-12, EJ, OJ and Ro52—but there was no evidence of seropositivity, excluding development of antibodies toward common myositis antigens and suggestive of T cell-mediated toxicity.

### Analysis of T cell repertoires

**Figure 2:**
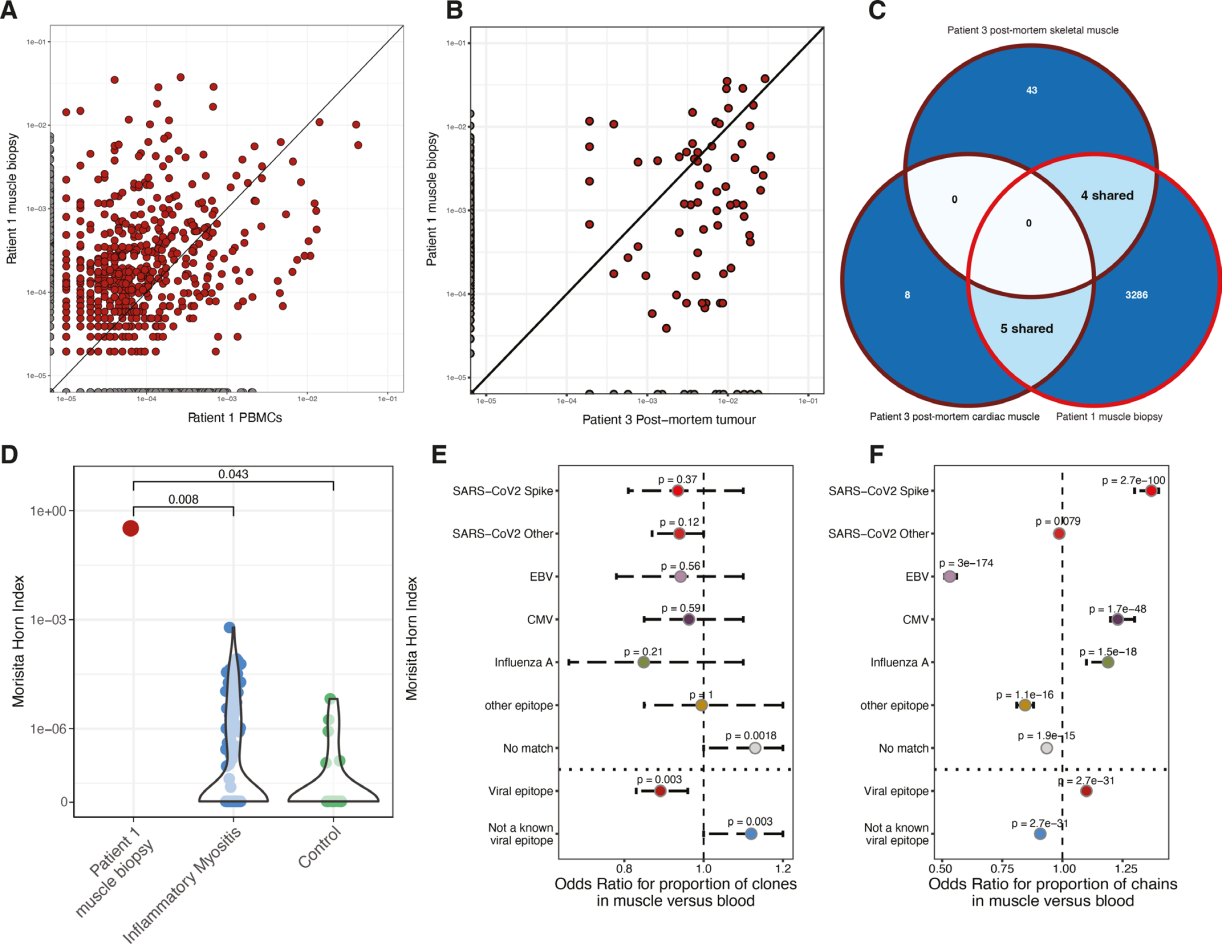
(A) Dot plot showing proportion of repertoire occupied by T cell clone in peripheral blood versus muscle biopsy from patient 1. (B) As for (A) but comparing the tumor from patient 3 with the muscle from patient 1. (C) A Venn diagram of the TCR overlap between cardiac and skeletal muscle of patient 3 and skeletal muscle of patient 1. (D) Morisita-Horn (MH) index for the repertoire overlap between the resected tumor from patient 3 and the skeletal muscle of patient 1, and samples taken from patients with IIM.^18^ (E) OR for occurrence of epitope-specific clones (TCR) found in the muscle versus the peripheral blood of patient 1. (F) As per (E) but taking into account number of copies of each clone (thereby considering clonal expansion). Statistics are via the Kolmogorov-Smirnov test (D) or Fisher’s exact test (E, F). IIM, idiopathic inflammatory myositis.

TCR sequencing (TCRseq) of peripheral blood and tissue samples from all three patients (detailed in online supplemental table S1) was performed, identifying a high degree of clonal sharing both within individuals (different tissue sites) and between individuals (figure 2, online supplemental figures S1 and S2). Clonally expanded TCRs present in the muscle biopsy from patient 1 were found similarly expanded within the peripheral blood, with a trend toward greater muscle expansion (figure 2A). Notably, there was high clonal overlap between TCR found in the PM tumor specimen from patient 3 and the other sampled tissue sites (figure 2, online supplemental figure S2). Of the 13 TCR found within the PM cardiac muscle of patient 3, 5 matched those found within the skeletal muscle biopsy of patient 1 (figure 2C) and one was found in the peripheral blood of patient 2 (not shown).

To quantify repertoire overlap, we applied the Morisita-Horn (MH) similarity index.^17^ This confirmed high overlap between the TCR repertoire from the PM tumor sample from patient 3 and the fresh muscle biopsy from patient 1 (MH 0.328) (figure 2D). This was significantly greater than the overlap between the PM tumor and TCR repertoires sequenced from muscle biopsies taken from controls and patients with idiopathic inflammatory myositis (IIM)^18^ (median MH for IIM samples 0, IQR 0–8.72e−7, p=0.008) (figure 2D), further illustrated by plotting clonal sharing (online supplemental figure S3). We explored the overlap of the TCR repertoires from resected melanomas from eight patients in our original cohort (all resections pre-2020), (online supplemental table S2). There was limited overlap and clonal sharing between these tumors and the muscle of patient 1, which was the same as their overlap with IIM samples (online supplemental figures S4 and S5A). A similar finding was noted when comparing the TCR found within the cardiac tissue of patient 3 and either IIM muscle biopsies or previously resected tumors (online supplemental figures 5B,C).

Finally, we examined the nature of putative TCR epitopes from specimens taken from patient 1, with multiple shared clones matching a range of viral epitopes (online supplemental table S3). We tested for the enrichment of each clone within the muscle versus the peripheral blood, finding that although no significant differences in total unique clones recognizing specific epitopes (figure 2E), when taking into account clone size, TCR putatively recognizing the SARS-CoV2 Spike protein were enriched within the muscle (OR 1.37, 95% CI 1.30 to 1.40, p<0.0001), along with TCR reactive to viral epitopes in general (figure 2F).

## Discussion

We describe a cluster of three highly unusual cases presenting with clinically significant and life-threatening acute myositis with cardiac involvement within a 3-week window, postreceipt of the first 6 weekly dosage of pembrolizumab. Pharmacovigilance studies of immunotherapy-associated myocarditis show a higher incidence and increased severity in recipients of combination anti-CTLA-4 and anti-PD-1 immunotherapy, while approximately 25% of cases show evidence of myositis and 10% have features of myaesthenia gravis.^19^ Occurrence of all three is referred to as the ‘3M syndrome’, has a much lower incidence than myocarditis alone, and is more severe.^20^ Strikingly, all three described cases had characteristics of the ‘3M syndrome’. None of the attending physicians had witnessed such rapid-onset and severe myositis with respiratory, cardiac and bulbar involvement post-ICB over many previous years of practice, underlining the highly unusual occurrence of three cases within 22 days in the same institute.

Analysis of TCR sequencing reveals expansion of similar clones across multiple samples. The similarity between TCR found in the PM cardiac muscle, skeletal muscle and tumor of patient 3 and the skeletal muscle of patient 1 is significantly higher than when compared with the TCR repertoires sequenced from muscle biopsies of IIM or than seen between these tissues and resected melanomas from pre-2020. As such, this suggests clonal expansion of a subset of public clones not found in other forms of myositis, indicating possible recognition of a distinct set of antigens common to both muscle and tumor. Some of these TCR are known to be Spike-reactive, and muscle-infiltrating TCR are enriched for this epitope compared with the peripheral blood of the same patient. Cross-reactivity between melanoma and muscle antigens is a previously described phenomenon with MAGE-A3-specific TCR known to react against titin in cardiac muscle, with fatal consequences^21^ and promiscuity of melanoma reactive TCR is increasingly recognized.^22^ There are a limited number of published titin-reactive TCR (two in public datasets), which were not identified in our study. Nonetheless, the high degree of clonal sharing between the melanoma deposit in patient 3 and the muscle specimens adds credence to this theory and it may be that there is a greater overlap between melanoma and muscle antigens than previously anticipated.

Myocarditis following ICB therapy is well described,^5 23 24^ as is an association with mRNA COVID-19 vaccines.^9–12^ Cardiomyocytes express high levels of PD-L1 which is upregulated in the context of myocardial injury, serving to abrogate severe myocarditis.^25–29^ Similarly, PD-L1 expression in the inflamed tumor microenvironment curtails antitumor T cell activity. Following injection of Spike mRNA into muscle, it is primarily myocytes that express the antigen and the immune response is, therefore, directed against the muscle cells themselves. The local inflammation and CD8+-associated response have the potential to release muscle antigens into the tissue microenvironment and inadvertently elicit a degree of antimuscle immune activity. In the context of subsequent early anti-PD1 treatment, the physiological feedback through PD-L1:PD-1 ligation and peripheral tolerance mechanisms preventing development of systemic autoimmunity may be overcome. We postulate these cases may represent the consequence of de novo anti-PD1 infusion postboost vaccination, revealing antimuscle autoimmunity with concomitant myaesthenia gravis symptoms.

An increase in incidence of myocarditis and myositis post-ICB has been noted by others since the beginning of the COVID-19 pandemic,^30 31^ and it is possible similar cases with this unusual clinical presentation have been overlooked. Further, multiple case series describing other irAEs have been recently published as summarized in a recent review^32^ and toxicity has been noted postvaccination as well as following natural infection.^33^ Further, an overall increase in incidence of autoimmune diseases following the pandemic has been described.^34^ Together, there is a growing corpus of circumstantial evidence to suggest immunological interplay between COVID-19, the vaccination and ICB may increase risk of severe toxicities, including irAEs previously felt to be extremely rare, such as ‘3M syndrome’.

Notably, these data are observational and causality is not assigned. Moreover, the TCRseq data are based on CDR3B amino acid sequence rather than the complete chain. We suspect the high number of putative Spike-recognizing TCR reflects, at least in part, extensive prior analysis of this epitope and disproportionate representation in databases. In keeping with this, there was a generalized enrichment of TCR recognizing antiviral epitopes. Finally, we have recently described the association of the minor allele of rs16906115, intragenic to *IL7*, with the development of IRAEs to ICB.^35 36^ We tested the three index cases for carriage of this allele but found all to be homozygous for the major allele, arguing against common genetic predisposition and again indicating a recent shared environmental factor.

Any potential clinical risk identified by this study needs to be considered in the context of the significant benefits COVID-19 vaccination provides, especially in patients with cancer. The literature supports the role of COVID-19 vaccination in patients with cancer already receiving ICB^37^ with higher seroconversion rates noted compared with patients those on chemotherapy.^38 39^ Nonetheless, in addition to the temporal clustering of these cases over a 2-month period, these cases are all characterized by the infusion of a 42-day (high) dose of pembrolizumab in a 12-week window postbooster vaccination with toxicities developing prior to any subsequent infusions. In order to mitigate risk, we would urge physicians to be aware of this association, with a low threshold for assessment and monitoring of ECG changes, blood enzymes and clinical status. Consideration should be given to initiating 3-weekly pembrolizumab prior to switching after four cycles and booster vaccination being given postinitiation of ICB treatment as opposed to in the prior weeks. Finally, due to the overall low incidence of these toxicities, any adverse events should be reported to regulators via usual channels so that population-level monitoring can continue.
