# Supplementary material for: Severe acute myositis and myocarditis on initiation of 6-weekly pembrolizumab post-COVID-19 mRNA vaccination
Source: J Immunother Cancer. 2024 Apr 24;12(4):e008151. doi: 10.1136/jitc-2023-008151 (PMC11043765; doi:10.1136/jitc-2023-008151)
Supplement: Supplementary data [file jitc-2023-008151supp001.pdf]

JITC-2023-008151.R1

Supplemental information

Supplemental Tables

Reported (index) cases		Myositis controls (Montagne) - Fresh Frozen Muscle Biopsies
Patient 1	PBMCs (post treatment)	IMNM
	Fresh muscle biopsy (post treatment)	DM
Patient 2	PBMCs (post treatment)	IBM
Patient 3	PBMCs (pre treatment)	ASyS
	PM heart (post treatment)	Non-IIM
	PM muscle (post treatment)	Healthy
	PM tumour (post treatment)	

Table S1. Samples on which TCRseq was performed. PBMC - peripheral blood mononuclear cell; PM - post mortem; IMNM - immune-mediated necrotising myositis; DM - dermatomyositis; IBM - inclusion body myositis; AyS - anti synthetase syndrome; IIM - idiopathic inflammatory myositis. Myositis control samples came from Montagne *et al*[18]

Tumour block	Specimen resection date	Specimen detail
T51	19/01/2017 x2	Excised melanoma from skin (x2 lesions)
T61	22/02/2017	Excised melanoma from skin
T64	07/04/2017	Melanoma deposit within lymph node
T90	10/03/2017	Melanoma deposit within lymph node
T101	19/02/2018	Excised melanoma from skin
T104	23/02/2017,12/05/2017, 04/01/2018	Excised melanoma from skin (x1 lesion) and melanoma deposit within lymph node (x2 lesions
T108	27/06/2017	Excised melanoma from skin
T132	07/08/2018	Excised invasive melanoma from muscle

Table S2. Resection date and pathological detail for 12 resected melanoma specimens. Slides were microscopically examined to identify areas of melanoma before macrodissection and deparafinisation. TCR sequencing was then performed as described in Methods.

Table S3. THIS IS A CSV FILE. Chain information for patient 1 PBMC and muscle biopsy with IEDB organism, epitope and score.

Supplemental Figures

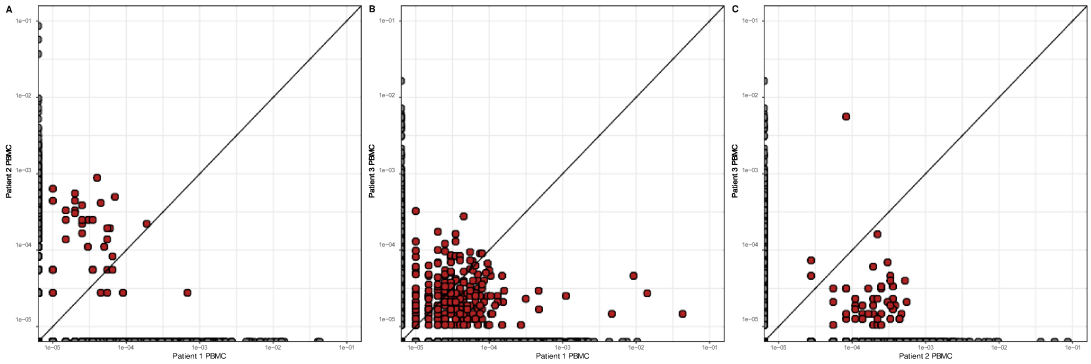

Figure S1. (A-C) Dot plot showing proportion of repertoire occupied by T cell clones in peripheral blood samples (as labelled).

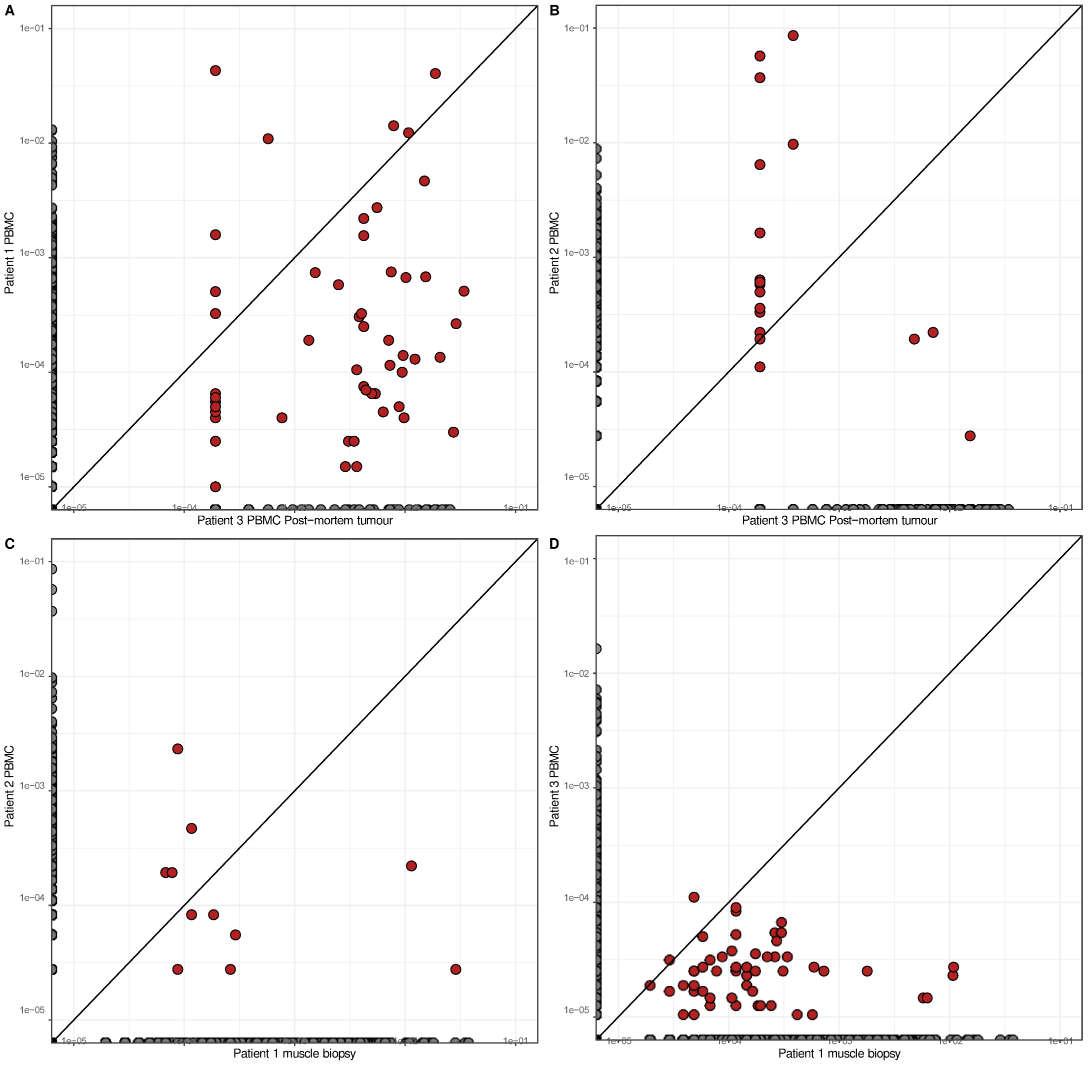

Figure S2. (A-B) Dot plot showing proportion of repertoire occupied by T cell clones in PM tumour sample from patient 3 compared to peripheral blood of patients 1 and 2 (as labelled). (C-D) as for (A-B) but comparing fresh muscle biopsy from patient 1 to peripheral blood of patients 2 and 3 (as labelled).

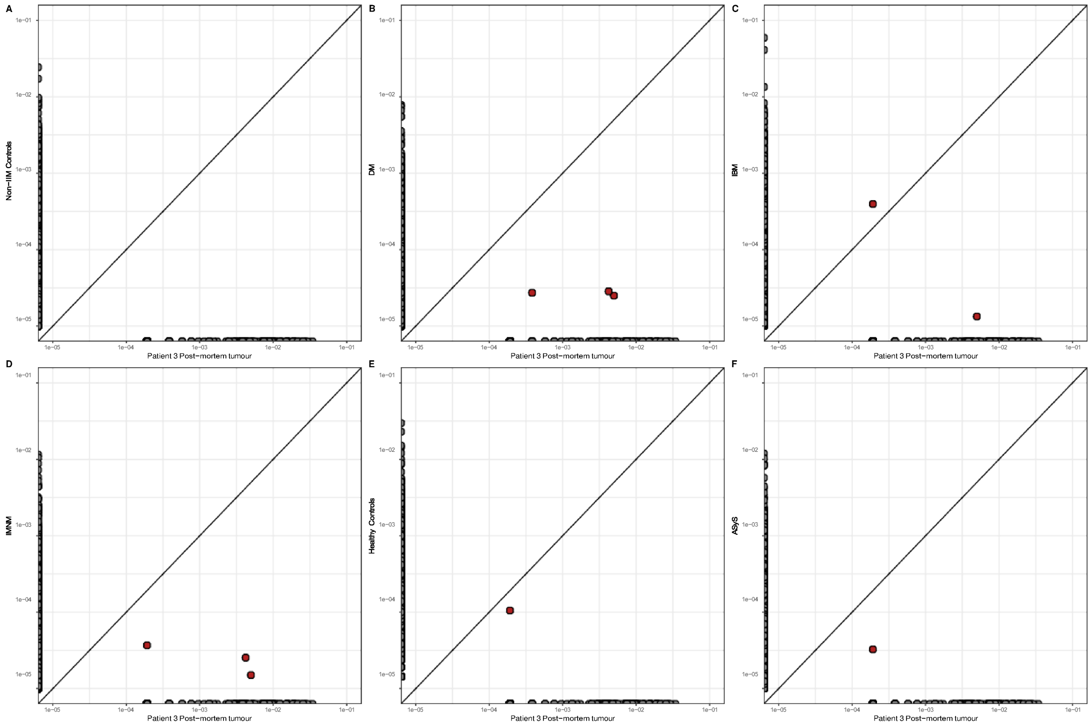

Figure S3. (A-F) Dot plot showing proportion of repertoire occupied by T cell clones in PM tumour sample from patient 3 compared to those found in muscle biopsies taken from patients with IIM[18]. N.B. patients with IIM are grouped by condition, each condition containing multiple individuals. IMNM - immune-mediated necrotising myositis; DM - dermatomyositis; IBM - inclusion body myositis; AyS - anti synthetase syndrome; IIM - idiopathic inflammatory myositis.

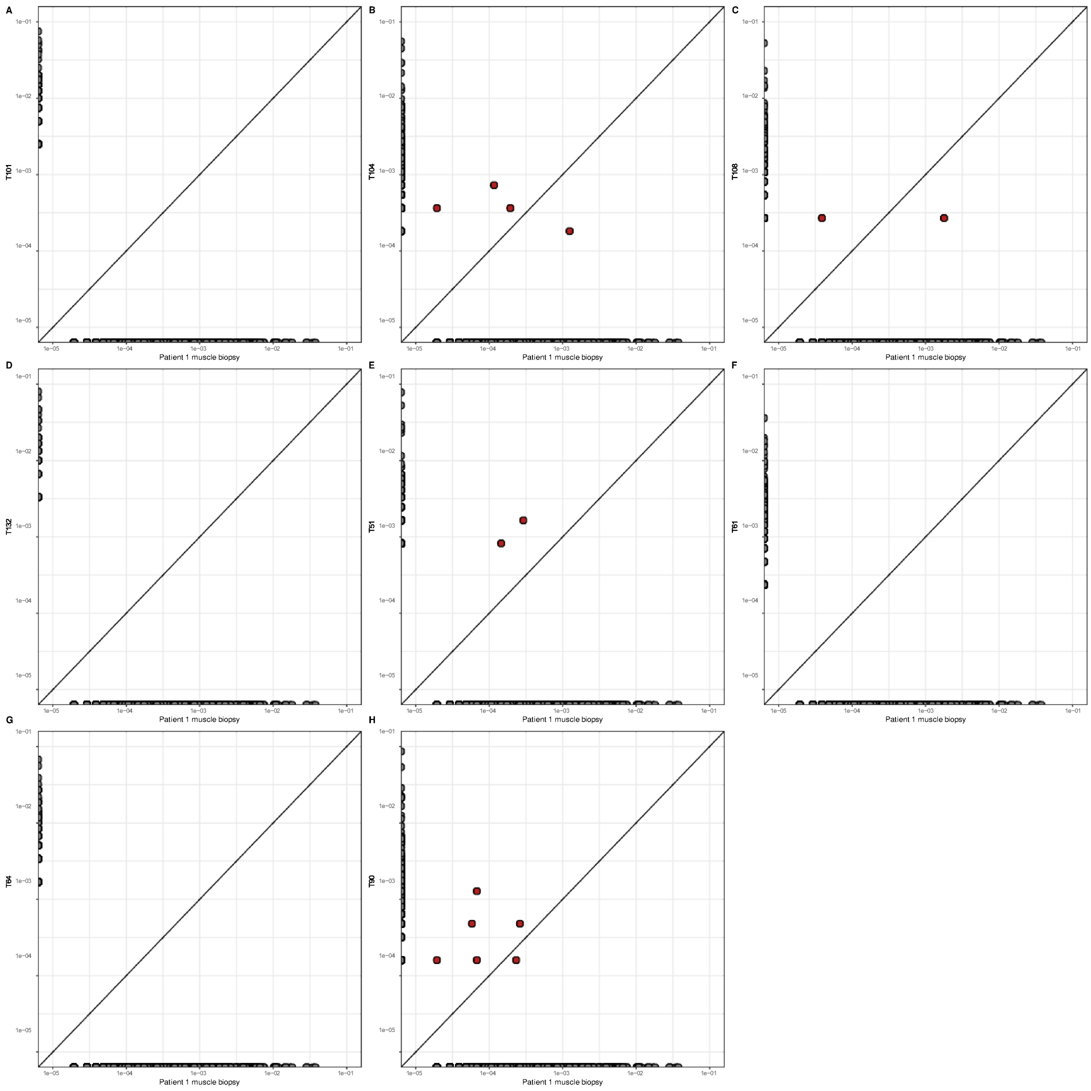

Figure S4. (A-H) Dot plot showing proportion of repertoire occupied by T cell clones in fresh muscle biopsy from patient 1 compared to those found in resected melanomas from before 2020.

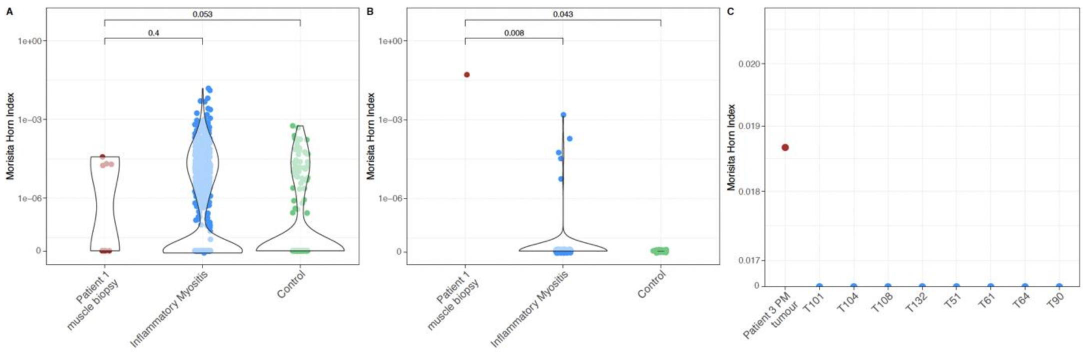

Figure S5. (A) Morisita-Horn (MH) index for the TCR repertoire overlap between resected melanomas from eight patients from the original cohort (table S2) and skeletal muscle biopsies as labelled (N.B. repertoires found in inflammatory myositis biopsies and control biopsies came from Montagne *et al*[18]). (B) as for (A) but MH index for TCR repertoire overlap between PM cardiac muscle from patient 3 and muscle biopsies as labelled. (C) MH index showing TCR repertoire overlap between the PM cardiac muscle from patient 3, the PM tumour from patient 3 and resected melanomas from eight patients pre-2020 (table S2).

Supplemental Data

Supplementary report 1 – full histopathology report of skeletal muscle biopsy from patient 1

MICROSCOPIC REPORT:

Frozen sections show skeletal muscle with a significant increase in the variability of muscle fibre diameters (approximate range: 11- 96 μm). There are atrophic and some hypertrophic fibres. The main pathology is patchy and characterised by prominent multifocal clusters of pale necrotic and basophilic regenerating fibres associated with loose macrophage-rich inflammatory infiltrates. There is a focal increase in internal nuclei, which is likely secondary. There is a very focal increase in endomysial connective tissue associated with the areas of necrosis / regeneration. The blood vessels appear unremarkable. Paraffin-embedded material adds no further information.

Special Stains

Modified Gomori trichrome shows occasional vacuolated fibres, which appear to be necrotic. There are no convincing rods or ragged red fibres. The overall lipid content of muscle fibres is within normal limits. Rare fibres show a significant increase (significance uncertain). The glycogen content of muscle fibres is not increased. Oxidative enzyme analysis shows a small number of cytox negative / SDH positive fibres (likely secondary). There are no cores or targets.

Immunohistochemistry

The fast (type 2) fibres comprise 60-70% of fibres. There is clustering but no significant grouping of fibre types. Atrophic fibres are of both types. Scattered fibres (mostly atrophic) are positive for fetal myosin, a proportion of which are also positive for embryonic myosin consistent with regeneration. A significant number of fibres are positive for NCAM. There is strong patchy sarcolemmal and sarcoplasmic upregulation of MHC-1. LCA and PGM-1 highlight the macrophage-rich inflammatory infiltrates associated with clusters of necrosis / regeneration. C5b-9 stains necrotic fibres diffusely, as expected. In addition, there is capillary deposition. p62 shows a spectrum of diffuse and granular staining in a small number of fibres (non-specific). Spectrin highlights the variability of fibre diameters.

COMMENT: The clinical diagnosis of pembrolizumab-associated myositis is noted. Pembrolizumab (monoclonal antibody, immune checkpoint (PD-1) inhibitor) was administered as part of systemic melanoma therapy. This muscle biopsy shows features which are entirely supportiveof immune checkpoint inhibitor-associated myositis (ICIAM), as described in the literature (e.g. Shelly et al Brain Communications 2020, doi:10.1093/braincomms/fcaa181). In particular, the multifocal clusters of necrotic fibres as opposed to single scattered necrotic / regenerating fibres typically observed with immune-mediated necrotising myopathies (IMNM) are a characteristic finding in ICIAM. Cytox negative SDH positive fibres have also been described as a secondary feature. Close correlation with the ongoing clinical and any other relevant findings is advised.

DIAGNOSIS: MUSCLE BIOPSY - FEATURES SUPPORTIVE OF IMMUNE CHECKPOINT INHIBITOR (PEMBROLIZUMAB) - ASSOCIATED MYOSITIS (ICIAM). PLEASE SEE COMMENT.

Supplementary report 2 – histopathology report from post mortem examination of patient 3

HISTOLOGY HEART - There are multiple foci of inflammation and myocyte necrosis throughout the sampled cardiac tissue. The inflammatory cell population is predominantly composed of lymphocytes, plasma cells and macrophages. The lack of replacement fibrosis indicates the process has occurred within a two week timeframe.

SKELETAL MUSCLE - There are multiple foci of inflammation and skeletal muscle necrosis in skeletal muscle obtained from the proximal thigh and iliopsoas with similar appearances to the sampled cardiac muscle.

SMALL BOWEL - There are deposits of malignant neoplasm on the serosal surface of the small bowel which have a malignant spindle cell morphology with a high mitotic rate and patchy areas of brisk immune response. The appearances are in keeping with metastatic melanoma.

LUNGS - The lung tissue demonstrates

congestion. LIVER - The liver demonstrates

normal appearances.

KIDNEY - The kidney demonstrates endocapillary hypercellularity within glomeruli predomi- nantly composed of mononuclear cells. There is glomerulomegaly, mild interstitial fibrosis and tubular atrophy, and moderate arteriosclerosis.

SUMMARY Post mortem examination has confirmed the clinical suspicion of both myositis and myocarditis. There is extensive involvement in the sampled cardiac tissue and skeletal muscle with established myocyte necrosis. We note from the clinical team that intravenous methylprednisolone was given for three days, and a echocardiogram demonstrated preserved biventricular function. There was a rapid clinical deterioration to type 2 respiratory failure with intensive care discussion. In our opinion, this would favour a synergistic progression of both the confirmed cardiac myositis and the skeletal muscle myositis to account for the rapid clinical decline. The skeletal muscle myositis would have placed [patient] at risk of respiratory failure due to the lack of skeletal accessory muscle power aiding respiration. There is congestion present within the sampled lung tissue, but no additional pathology such as bronchopneumonia, pulmonary emboli, acute lung injury or organising pneumonia / fibrosis. We have histological evidence of the small volume metastatic melanoma on the serosal surface of the sampled small bowel: there are areas of brisk lymphocytic infiltration within the tumour which may represent treatment effect. The pattern of myositis and myocarditis is likely to be due to an autoimmune phenomenon precipitated by the immune-modulatory therapy for low volume metastatic melanoma. We understand that there are clinical concerns that this may be related to administration of a COVID-19 vaccine booster shortly before commencing therapy with pembrolizumab. Myocarditis has been described as a rare complication following the administration of COVID-19 vaccines with a frequency of 10 per million. To our knowledge, fatal myocarditis has not been described in the context of

pembrolizumab use following booster vaccine administration. We understand from the clinical team that this concern has already been flagged using the MHRA yellow card system.
